# The effect of intracranial pressure monitoring on severe traumatic brain injury patients who undergo subdural hematoma evacuation

**DOI:** 10.1007/s10143-026-04341-7

**Published:** 2026-05-23

**Authors:** Cade McGarvey, Aditya Acharya, Gang Ren, Jason Schroeder, Alastair Hoyt

**Affiliations:** 1 University of Toledo College of Medicine and Life Sciences, 3000 Arlington Ave., Toledo, OH 43614 USA; 2 Department of Neurosurgery, University of Toledo Medical Center, 3000 Arlington Ave., Toledo, OH 43614 USA

**Keywords:** Trauma, Intracranial pressure, Traumatic brain injury, Subdural hematoma, Monitor

## Abstract

Intracranial pressure (ICP) monitoring is frequently employed for patients with severe traumatic brain injury. The effect of intracranial pressure monitoring on patient outcomes has been studied little, and results often fail to account for confounding variables. We sought to focus on the impact of ICP monitoring on patients with severe traumatic brain injury and subdural hematoma (SDH) that underwent surgical intervention. Using the National Trauma Data Bank, we identified patients between 2021 and 2024 who had SDH and a presenting Glasgow Coma Score (GCS) of 3–8 who underwent an SDH evacuation. Of the patients whose ICP monitoring status was known, we compared the in-hospital mortality rate and length of stay (LOS) of patients who had ICP monitoring and those who did not. 3932 patients met the inclusion criteria, with 1481 patients undergoing ICP monitoring and 2451 not undergoing ICP monitoring. After propensity score matching to control for confounding variables, 1271 patients from each cohort were selected for further analysis. In-hospital mortality rate of the patients with monitoring did not show a significant difference from the cohort without ICP monitoring, in either matched (41.6% vs. 41.9%, p = 0.9) or without matching scenario (40.2% vs. 42.6%, p = 0.15). However, patients with ICP monitoring showed a longer hospital stay compared to the cohort without ICP monitoring (median 19 days vs. 13 days, p < 0.001). While ICP monitoring is recommended in brain injury treatment guidelines, it did not show a significant impact on the in-hospital mortality rate of patients with SDH and GCS 3–8 who had undergone SDH evacuation. Patients with ICP monitoring had significantly longer LOS and a higher rate of complications, including ventilator-associated pneumonia, deep venous thrombosis, and acute respiratory distress syndrome. Additional study is needed to determine if ICP monitoring improves outcomes in various populations of traumatic brain injury patients. Clinical trial number: not applicable.

**Supplementary Information:**

The online version contains supplementary material available at 10.1007/s10143-026-04341-7.

## Introduction

Traumatic brain injuries (TBI) are the cause of approximately 2.5 million visits to the emergency department and approximately 60,000 deaths annually [1]. Subdural hematoma (SDH) results from tearing of the bridging veins and often occurs due to TBI [2, 3]. The primary goal of ICP monitoring in cases of severe traumatic brain injury (sTBI) is to avoid intracranial hypertension, which can cause herniation of the brain, decreased perfusion, and deformation of tissue [1, 4, 5]. Surgical evacuation, in the form of craniectomy or craniotomy, is a standard treatment for SDH, but it is not indicated for every patient [3, 6–8].

There is a lack of data showing how ICP monitoring affects mortality rates and LOS, specifically in patients who have undergone SDH evacuation. This study aims to utilize International Classification of Diseases, Tenth Revision, Clinical Modification (ICD-10-CM) codes through the American College of Surgeons Trauma Quality Improvement Program (ACS TQIP) database to further understand the role of ICP monitoring in the context of subdural hematomas, which are a common cause of TBIs.

## Methods

### Database

This is a retrospective cohort study performed with the ACS TQIP database, which was obtained from the National Trauma Data Bank (NTDB) between 2021 and 2024. The ACS TQIP database is the largest aggregation of US trauma registry data ever assembled, which collects injury data from more than 900 trauma centers in the USA. The dataset includes de-identified records and is exempt from local board review.

### Selection criteria and data collection

Adult patients (age > = 18) with severe traumatic brain injury and subdural hematoma (SDH) that underwent surgical intervention were identified by ICD-10-CM coding (S06.5), following the standard of ICD-10-CM. Glasgow Coma Scale scores of 3 to 8 were applied to identify severe brain injury patients in this study. To keep the mechanism of injury consistent, we only included patients with blunt trauma. Included patients also had to have cerebral edema (S06.1). We excluded patients who had not undergone surgical procedures of craniotomy and craniectomy (ICD-10-CM procedure codes, Supplementary Table 1), and further divided them into two cohorts, with or without ICP monitor placement during the hospital stay (ICD-10-CM codes 4A103BD, 4A107BD, 4A108BD). In this definition, an ICP monitor could be either a fiberoptic monitor or external ventricular drain. We further validated the study cohorts and excluded records with a discrepancy between the ICD identifier and the trauma registry, missing records, and records missing ICP monitor placement time. Data collected included demographics (age, gender, race, ethnicity), BMI (calculated based on height and weight), injury severity score (ISS), GCS scores, mechanism of injury, trauma type, primary payers, hospital information (bed size, teaching status, hospital type, trauma center levels), pre-hospital cardiac arrest, and transfusion. The in-hospital variables and outcome variables collected were ICU LOS, total hospital LOS, ICP hours, emergency department disposition, hospital disposition, and in-hospital mortality. Pre-admission comorbidities and in-hospital complications were also analyzed after propensity score matching of the two cohorts.

A subgroup analysis was performed on patients whose ER disposition was to the OR. Propensity score matching was used in the same way as for our primary analysis, and we measured the same outcomes.

### Statistical analysis

Univariate analysis was used to identify differences in baseline and outcome variables between patients with ICP monitor placement versus those without. Categorical variables were compared using the Chi-Square test. The Mann–Whitney U test was used to compare medians for continuous variables. Results were reported as numbers and percentages for categorical variables or medians and interquartile ranges (IQR) for continuous variables. Propensity score matching analysis was used to compare patients with or without ICP monitor placement. Patients were matched on age, gender, race, ethnicity, BMI, ISS, GCS, trauma type, hospital bed size, teaching status, hospital type, and trauma center levels, with 1:1 nearest-neighbor propensity score matching without replacement. The primary outcome was mortality, and secondary outcomes included hospital LOS, discharge information, and complications. All analyses were prepared by statistical software SPSS (version 30.0) and R (version 2024.12.1), and database-related work was done by SQL under “sqldf” R package (https://cran.r-project.org/web/packages/sqldf/sqldf.pdf). *P* < 0.05 is considered statistically significant, and a two-tailed test was applied to all the statistical analyses.

## Results

**Fig. 1:**
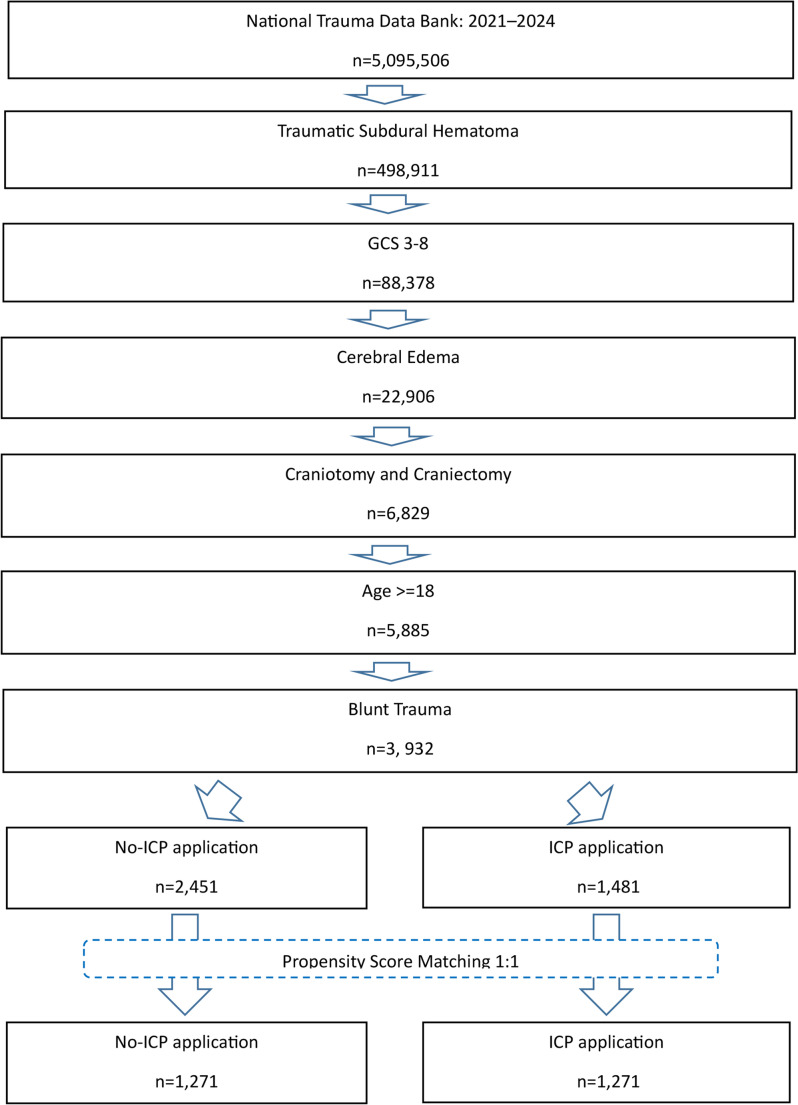
ICD-10-CM flowchart for analysis

Before propensity score matching, there were 3,932 patients 18 years or older between 2021 and 2024 who had a TBI with an SDH from blunt trauma, a presenting GCS of 3–8, cerebral edema, SDH evacuation, and had a known ICP monitoring status. There were 1,481 patients with an ICP monitor and 2,451 did not receive an ICP monitor (Fig. 1). The median age of the cohort was 43, with a gender distribution of 74.4% male and 25.3% female. Before propensity score matching, patients who underwent ICP monitoring were more likely to be younger than those without ICP monitoring (37 vs. 49 years (*p* < 0.001)), more likely to be male (77.4% vs. 72.6% (*p* < 0.001)), and more likely to have a higher ISS severity score (30 vs. 27 (*p* < 0.001)) (Table 1).

After 1:1 propensity score matching, cohorts of 1,271 patients with ICP monitoring and 1,271 patients without ICP monitoring were obtained. No significant demographic differences were found between the two groups. Patients with private insurance had significantly more ICP monitoring compared to patients with Medicare, Medicaid, or other methods of payment (*p* < 0.001). Patients with the comorbidities of anticoagulant therapy and congestive heart failure were less likely to receive ICP monitoring (3.5% vs. 7.9% (*p* < 0.001) and 0.8% vs. 3.1% (*p* < 0.001), respectively) (Table 1).

**Table 1:** Pre-matched patient characteristics

Demographic		All		nonICP		ICP		*P* value
		*n* = 3932		*n* = 2451		*n* = 1481		
Age, median(IQR)		43	(29, 62)	49	(32, 67)	37	(26, 51)	< 0.001
Age group	18–35	1093	27.8%	546	22.3%	547	36.9%	
	36–50	678	17.2%	376	15.3%	302	20.4%	
	51–65	584	14.9%	337	13.7%	247	16.7%	
	> 65	1577	40.1%	1192	48.6%	385	26.0%	
BMI, median(IQR)		26.3	(23.2, 30.3)	26.3	(23.0, 30.3)	26.4	(23.3, 30.4)	0.69
ISS, median(IQR)		29	(26, 35)	27	(25, 34)	30	(26, 38)	< 0.001
ISS severity	<=25 (moderate to severe)	476	12.1%	289	11.8%	187	12.6%	
	26–30 (very severe)	2331	59.3%	1571	64.1%	760	51.3%	
	> 30 Critical)	1124	28.6%	590	24.1%	534	36.1%	
GCS, median(IQR)		3	(3, 5)	3	(3, 6)	3	(3, 5)	0.22
GCS level	3	2601	66.1%	1605	65.5%	996	67.3%	
	4–6	750	19.1%	476	19.4%	274	18.5%	
	7–8	581	14.8%	370	15.1%	211	14.2%	
Gender	Male	2927	74.4%	1780	72.6%	1147	77.4%	< 0.001
	Female	996	25.3%	664	27.1%	332	22.4%	
Race	White	2621	66.7%	1655	67.5%	966	65.2%	0.14
	non-White	1311	33.3%	796	32.5%	515	34.8%	
Ethnicity	Hispanic or Latino	646	16.4%	366	14.9%	280	18.9%	0.002
Transfusion		1397	35.5%	828	33.8%	569	38.4%	0.003
Pre-Hospital Cardiac Arrest		133	3.4%	81	3.3%	52	3.5%	0.74
Trauma center level	Level 1	2340	59.5%	1395	56.9%	945	63.8%	< 0.001
	other	1592	40.5%	1056	43.1%	536	36.2%	
Mechanism of Injury	Fall	1610	40.9%	1217	49.7%	393	26.5%	< 0.001
	MVT/Transport accidents	1803	45.9%	924	37.7%	879	59.4%	
	Struck by and other	519	13.2%	310	12.6%	209	14.1%	
Bedsize	Small/Medium ( < = 600)	2361	60.0%	1448	59.1%	913	61.6%	0.11
	Large Hospital(> 600)	1571	40.0%	1003	40.9%	568	38.4%	
Hospital Teaching Status	Academic/university	2039	51.9%	1174	47.9%	865	58.4%	< 0.001
	Community/Nonteaching	1878	47.8%	1270	51.8%	608	41.1%	
Hospital Type	For Profit	481	12.2%	343	14.0%	138	9.3%	< 0.001
	Non-profit/government	3449	87.7%	2108	86.0%	1341	90.5%	
Payment	Private/Commercial Insurance	1502	38.2%	833	34.0%	669	45.2%	< 0.001
	Medicare	768	19.5%	644	26.3%	124	8.4%	
	Medicaid	878	22.3%	488	19.9%	390	26.3%	
	Self pay and Other	784	19.9%	486	19.8%	298	20.1%	

After 1:1 propensity score matching, cohorts of 1,271 patients with ICP monitoring and 1,271 patients without ICP monitoring were obtained. No significant demographic differences were found between the two groups. Patients with private insurance had significantly more ICP monitoring compared to patients with Medicare, Medicaid, or other methods of payment (p<0.001). Patients with the comorbidities of anticoagulant therapy and congestive heart failure were less likely to receive ICP monitoring (3.5% vs 7.9% (p<0.001) and 0.8% vs 3.1% (p<0.001), respectively) (Table 2).

**Table 2:** Propensity score matched patient characteristics

Demographic		nonICP		ICP		*P* value
		*n* = 1271		*n* = 1271		
Age, median(IQR)		38	(27, 57)	38	(27,53)	0.22
Age group	18–35	437	34.4%	426	33.5%	
	36–50	258	20.3%	255	20.1%	
	51–65	180	14.2%	225	17.7%	
	> 65	396	31.2%	365	28.7%	
BMI, median(IQR)		26.3	(23.0, 30.4)	26.4	(23.4, 30.4)	0.62
ISS, median(IQR)		30	(26, 38)	30	(26, 38)	0.34
ISS severity	<=25 (moderate to severe)	148	11.6%	169	13.3%	0.45
	26–30 (very severe)	692	54.4%	678	53.3%	
	> 30 Critical)	431	33.9%	424	33.4%	
GCS, median(IQR)		3	(3, 5)	3	(3, 5)	0.22
GCS level	3	874	68.8%	843	66.3%	
	4–5	223	17.5%	243	19.1%	
	6–8	174	13.7%	185	14.6%	
Gender	Male	992	78.0%	975	76.7%	0.42
	Female	279	22.0%	296	23.3%	
Race	White	858	67.5%	838	65.9%	0.40
	non-White	413	32.5%	433	34.1%	
Ethnicity	Hispanic or Latino	216	17.0%	226	17.8%	0.60
Transfusion		466	36.7%	476	37.5%	0.68
Pre-Hospital Cardiac Arrest		42	3.3%	41	3.2%	0.91
Trauma center level	Level 1	770	60.6%	783	61.6%	0.60
	other	501	39.4%	488	38.4%	
Mechanism of Injury	Fall	380	29.9%	380	29.9%	1.00
	MVT/Transport accidents	806	63.4%	806	63.4%	
	Struck by and other	85	6.7%	85	6.7%	
Bedsize	Small/Medium ( < = 600)	777	61.1%	792	62.3%	0.54
	Large Hospital(> 600)	494	38.9%	479	37.7%	
Hospital Teaching Status	Academic/university	701	55.2%	701	55.2%	1.00
	Community/Nonteaching	570	44.8%	570	44.8%	
Hospital Type	For Profit	134	10.5%	134	10.5%	1.00
	Non-profit/government	1137	89.5%	1137	89.5%	
Payment	Private/Commercial Insurance	497	39.1%	584	45.9%	< 0.001
	Medicare	198	15.6%	118	9.3%	
	Medicaid	307	24.2%	311	24.5%	
	Other	269	21.2%	258	20.3%	

Patients with an ICP monitor did not have a significantly different in-hospital mortality rate compared to patients without ICP monitoring (41.6% vs. 41.9% (*p* = 0.9)). However, the LOS in the hospital was longer for patients with ICP monitoring compared to those without (19 days with IQR 8–35 days vs. 13 days with IQR 5–26 days (*p* < 0.001)). The findings were similar for ICU LOS (14 days with IQR 7–23 days vs. 8 days with IQR 4–16 days (*p* < 0.001)). Patients with ICP monitoring are more likely to be discharged to a care facility or another hospital (28.6% vs. 23.0% (*p* = 0.001)) and less likely to be discharged home (5.4% vs. 9.2% (*p* < 0.001)). Patients with ICP monitoring were also more likely to have complications of Acute Respiratory Distress Syndrome (4.7% vs. 1.8% (*p* < 0.001)), deep surgical site infection (1.1% vs. 0.3% (*p* = 0.018)), deep venous thrombosis (9.0% vs. 3.9% (*p* < 0.001)), organ/space surgical site infection (1.1% vs. 0.2% (*p* = 0.007)), pulmonary embolism (3.6% vs. 0.9% (*p* < 0.001)), stroke/cva (5.4% vs. 3.1% (*p* = 0.003)), ventilator-associated pneumonia (13.5% vs. 7.0% (*p* < 0.001)), pressure ulcer (6.7% vs. 3.1% (*p* < 0.001)), and unplanned visit to the OR (16.4% vs. 8.1% (*p* < 0.001)). Patients without ICP monitoring had a significantly greater number of unplanned intubations (4.7% vs. 2.3% (*p* < 0.001)) (Table 3). Similar primary outcomes were found before propensity score matching (Supplementary Table 2).

**Table 3:** Patient outcomes

Outcome		Non-ICP		ICP		*P* value
ICU length of stay, days, median (IQR)		8	(4, 16)	14	(7, 23)	< 0.001
Length of stay, days, median (IQR)		13	(5, 26)	19	(8, 35)	< 0.001
ICP hours (median, IQR)				3.4	(2.05, 7.72)	
Emergency department disposition	Operating room	818	64.4%	644	50.7%	< 0.001
	ICU	422	33.2%	608	47.8%	< 0.001
Hospital disposition	Home/routine	117	9.2%	69	5.4%	< 0.001
	Inpatient rehab	303	23.8%	293	23.1%	0.64
	Care facility or other hospitals	292	23.0%	364	28.6%	0.001
Died in hospital		532	41.9%	529	41.6%	0.90
**Comorbidities**						
Current Smoker		203	16.0%	190	14.9%	0.51
Chronic Renal Failure		18	1.4%	12	0.9%	0.36
Diabetes Mellitus		108	8.5%	76	6.0%	0.018
Hypertension		236	18.6%	188	14.8%	0.012
Anticoagulant Therapy		100	7.9%	45	3.5%	< 0.001
Myocardial Infarction		5	0.4%	5	0.4%	1.00
COPD		29	2.3%	17	1.3%	0.10
Congestive Heart Failure		40	3.1%	10	0.8%	< 0.001
**Complications**						
Acute Kidney Injury		20	1.6%	27	2.1%	0.30
Acute Respiratory Distress Syndrome (ARDS)		23	1.8%	60	4.7%	< 0.001
Cardiac Arrest with CPR		59	4.6%	43	3.4%	0.11
Deep Surgical Site Infection		4	0.3%	14	1.1%	0.018
DVTHROMBOSIS		50	3.9%	115	9.0%	< 0.001
Organ/Space Surgical Site Infection		3	0.2%	14	1.1%	0.007
Pulmonary Embolism		12	0.9%	46	3.6%	< 0.001
Stroke / CVA		39	3.1%	69	5.4%	0.003
Unplanned Intubation		60	4.7%	29	2.3%	< 0.001
Unplanned Admission to the ICU		64	5.0%	72	5.7%	0.48
Severe Sepsis		18	1.4%	28	2.2%	0.14
Catheter-Associated Urinary Tract Infection		8	0.6%	10	0.8%	0.64
Ventilator-Associated Pneumonia		89	7.0%	172	13.5%	< 0.001
Pressure Ulcer		40	3.1%	85	6.7%	< 0.001
Unplanned Visit to OR		103	8.1%	209	16.4%	< 0.001

In our subgroup analysis, there were 2,306 patients whose ER disposition was to the OR (744 received ICP monitoring and 1,562 did not) (Supplementary Table 3). After 1:1 propensity score matching, 665 patients received ICP monitoring and 665 did not (Supplementary Table 4). The results showed no significant difference in the in-hospital mortality rate (46.0% vs. 45.9% (*p* = 0.96)). The LOS for both the hospital and ICU was longer for patients with ICP monitoring (16 days with IQR 7–31 vs. 11 days with IQR 4–24 (*p* < 0.001) and 12 days with IQR 6–21 vs. 7 days with IQR 3–14 (*p* < 0.001), respectively). Patients with ICP monitoring were more likely to be discharged to a care facility or other hospital (26.5% vs. 20.2% (*p* = 0.006)) but were less likely to be discharged home compared to patients without ICP monitoring (5.4% vs. 9.8% (*p* < 0.001)) (Table 4).

**Table 4:** Subgroup analysis of patient outcomes

Outcome		Non-ICP		ICP		*P* value
ICU length of stay, days, median (IQR)		7	(3, 14)	12	(6, 21)	< 0.001
Length of stay, days, median (IQR)		11	(4, 24)	16	(7, 31)	< 0.001
ICP hours (median, IQR)				2.73	(1.77, 6.22)	
Hospital disposition	Home/routine	65	9.8%	36	5.4%	< 0.001
	Inpatient rehab	153	23.0%	142	21.4%	0.47
	Care facility or other hospitals	134	20.2%	176	26.5%	0.006
Died in hospital		305	45.9%	306	46.0%	0.96
**Comorbidities**						
Current Smoker		112	16.8%	94	14.1%	0.17
Chronic Renal Failure		8	1.2%	8	1.2%	1.00
Diabetes Mellitus		62	9.3%	40	6.0%	0.02
Hypertension		116	17.4%	98	14.7%	0.012
Anticoagulant Therapy		61	9.2%	33	5.0%	0.003
Myocardial Infarction		1	0.2%	3	0.5%	0.63
COPD		17	2.6%	12	1.8%	0.35
Congestive Heart Failure		21	3.2%	4	0.6%	< 0.001
**Complications**						
Acute Kidney Injury		14	2.1%	13	2.0%	0.85
Acute Respiratory Distress Syndrome (ARDS)		18	2.7%	29	4.4%	0.10
Cardiac Arrest with CPR		40	6.0%	17	2.6%	0.002
Deep Surgical Site Infection		2	0.3%	10	1.5%	0.02
DVTHROMBOSIS		20	3.0%	56	8.4%	< 0.001
Organ/Space Surgical Site Infection		2	0.3%	4	0.6%	0.69
Pulmonary Embolism		5	0.8%	26	3.9%	< 0.001
Stroke / CVA		18	2.7%	36	5.4%	0.012
Unplanned Intubation		18	2.7%	13	2.0%	0.36
Unplanned Admission to the ICU		29	4.4%	34	5.1%	0.52
Severe Sepsis		12	1.8%	12	1.8%	1.00
Catheter-Associated Urinary Tract Infection		4	0.6%	6	0.9%	0.53
Ventilator-Associated Pneumonia		43	6.5%	69	10.4%	0.01
Pressure Ulcer		18	2.7%	32	4.8%	0.044
Unplanned Visit to OR		42	6.3%	76	11.4%	< 0.001

## Discussion

A large majority of patients with SDH evacuation did not receive ICP monitoring (2,451 vs. 1,481). Postoperative care of brain injury patients varies according to the individual surgeon and patient. Factors like age and severity of injury can impact surgeon choices. Before propensity score matching, there were significant differences in demographics, ISS severity, hospital setting, and mechanism of injury between those who received ICP monitoring and those who did not. After propensity score matching, the confounders were well controlled. However, it was notable that patients with private insurance were more likely to receive ICP monitoring. Patients with anticoagulant therapy and congestive heart failure were less likely to have received ICP monitoring. In this study, propensity score matching attempted to control for several of the available confounding factors, but surgeon preference and experience may still produce bias.

The primary outcomes were in-hospital mortality and LOS. There was no significant difference in the in-hospital mortality between the group with ICP monitoring and the group without it, consistent with the BEST TRIP results. The LOS in both the hospital and ICU was significantly longer for the group with ICP monitoring, which differs from the BEST TRIP findings. ISS and GCS are not significantly different between the two groups [9]. Primary outcomes and disposition were similar in the subgroup analysis.

The BEST TRIP trial is currently the only randomized controlled trial that tested TBI patient outcomes with ICP monitoring. In that trial, it was found that there was no significant difference in the survival time, 3- and 6-month functional status, impaired consciousness, ICU length of stay (LOS), or 6-month mortality rate between patients with and without ICP monitoring. However, the study has been heavily criticized due to its inclusion of pediatric patients and the study site was in South American countries with significantly different healthcare infrastructures compared to the U.S [9]. Further work has been done in retrospective reviews regarding when ICP monitoring is employed, and outcomes of patients who receive ICP monitoring. One study found that between 2013 and 2017, 21,374 patients recorded in the National Trauma Data Bank had sTBI, and only 6543 of these patients received ICP monitoring, meaning almost 70% of sTBI cases did not receive ICP monitoring [4]. Another study focused on moderate traumatic brain injuries (mTBI) between 2015 and 2021 with a GSC score between 9 and 11. It found that ICP monitoring was associated with better clinical outcomes six months after discharge and a lower incidence of neurological deterioration for seven days post-injury [10]. LOS is a common measure in comparing ICP monitored and non-monitored TBI patient populations. One retrospective study in a level II trauma center found that ICP monitoring was associated with longer LOS, ventilator days, and a higher rate of pneumonia. However, our data differed in discharge disposition in that we found a significantly higher percentage of patients without ICP monitoring were discharged home compared to patients with ICP monitoring. In their study, patients with ICP monitoring were more likely to have a discharge disposition to home, but the data failed to reach statistical significance (*p* = 0.06) [11]. Because the database did not report GCS Extended or Modified Rankin Scale, disposition was used as a surrogate for neurological outcome.

Despite the secondary effects of ICP monitoring, one study was able to identify a very clear use of ICP monitoring. In the case of acute subdural hematoma, the first objective is to reduce intracranial pressure. This study found that placing an ICP monitor before burr hole surgery allowed for immediate relief of ICP, preventing herniation. The ICP monitor could then be used to detect ICP for the next three hours, as this window of time is when the brain is undergoing traumatic coagulopathy and any further surgery could lead to hemorrhage. ICP monitoring was used to ensure ICP did not elevate to dangerous levels. After that three-hour window, craniotomy could be safely performed without risk of hemorrhage. This specific use of ICP monitoring in the case of ASDH suggests its usefulness in monitoring patient condition while the patient is being stabilized [12].

Prior data have been conflicting regarding the mortality rate between patients with and without ICP monitoring. Many studies show no difference in mortality rates [13–15] while others show lower in-hospital mortality with ICP monitoring [1, 16, 17] and some show a higher mortality rate with ICP monitoring [18]. Such variation indicates that additional factors not previously studied contribute to the conditions in which ICP monitoring is more likely to improve patient outcomes. Here, we isolated patients with an SDH amenable to surgical evacuation, which also excluded patients for whom surgical evacuation would have been futile.

ICP monitoring has been associated with increased LOS in many studies [1, 11, 13, 14]. There have also been some which found no significant difference in LOS based on ICP monitoring status [19, 20] and Chen et al. found ICP monitoring decreased ICU LOS [17]. Our findings were consistent with the first set of studies and are intuitive in that measuring and responding to ICP readings likely results in increased treatment. However, our subgroup analysis showed that ER disposition did not affect outcomes, so the initial decision to use surgical treatment was not affected by ICP monitoring.

Some prior work investigated how the type of intracranial hemorrhage affected patient outcomes with TBI. Tang et al. in their retrospective single-institution study found a mortality benefit from ICP monitoring for SDH patients in the univariate analysis, but not in the multivariate analysis. They also found that only some subsets of patients had a decrease in mortality rate with ICP monitoring. That study examined all methods of ICP monitoring, encompassing both patients with and without surgical evacuation [21]. Alali et al. found ICP monitoring made no significant difference in mortality for SDH patients using the ACS TQIP database. However, patients with and without surgical evacuation were included, as well as some pediatric patients. All types of ICP monitoring were included. LOS was not a measured outcome [16].

Complications were higher in patients with ICP monitoring, specifically acute respiratory distress syndrome, deep surgical site infection, DVT thrombosis, organ/space surgical site infection, pulmonary embolism, stroke/cva, ventilator-associated pneumonia, pressure ulcer, and unplanned visit to the OR. The increase in complication rates is consistent with most literature, as this is an additional invasive procedure that patients undergo [11, 19, 22]. A higher rate of complications may also be a result of longer LOS, as more time is available for complications to arise. In our data, ISS and GCS were not significantly different between patients with and without ICP monitoring, so the severity of patient injuries was unlikely to have affected the complication rates we found. The one complication we found to be more prevalent in patients without ICP monitoring was unplanned intubations.

Therefore, without a demonstrated mortality benefit, an increased LOS, an increase in the complication rate, and no significant difference in the severity of the condition playing a confounding role, the results call into question the necessity of a blanket recommendation to monitor ICP in patients who underwent SDH evacuation and presented with a GCS 3-8 [21].

The results are from a retrospective review of the NTDB, not a randomized clinical trial, which limits the results. However, a randomized clinical trial on TBI with ICP monitoring presents many ethical problems, and when limited to only patients with SDH, it can make obtaining a sufficient sample size difficult, especially at a single institution. While the use of GCS for the inclusion criteria of patients is not perfect due to factors that can alter a patient’s GCS (e.g., intubation), it has been the standard methodology in similar work, including the BEST TRIP trial. While we could obtain GCS ranges for patients, we could not obtain individual GCSs for every patient due to ICD-10-CM coding limitations, which are also subject to misclassification. Using ICD-10-CM, we could not get longitudinal data on the patients. There is also reason to believe the type of ICP monitor used can affect patient outcomes [23]. Included codes included monitoring using any type of pressure monitoring device, including fiberoptic monitors and external ventricular drains. Because this is a retrospective review, causality could not be demonstrated, only a strong association. Causality is further limited by the inability of this study to define the temporal relationship between ICP monitoring and surgery.

## Conclusions

The results demonstrate that for patients in the U.S. with an SDH evacuation and a GCS 3–8, ICP monitoring is not associated with a significant difference in mortality rate, but it is associated with an increased LOS both in the hospital and in the ICU. ICP monitoring was also associated with significant increases in several complications, including ARDS, pneumonia, DVT, and PE. Additional patient populations should be studied to determine more specific conditions for which ICP monitoring would have the best indication, improving outcomes in patients with TBI. The effects of different types of ICP monitors on patient outcomes should also be studied.

## Supplementary Information

Below is the link to the electronic supplementary material.

Supplementary Material 1

## Data Availability

All data is available in the manuscript.
