# Supplementary material for: The effect of intracranial pressure monitoring on severe traumatic brain injury patients who undergo subdural hematoma evacuation
Source: Neurosurg Rev. 2026 May 23;49(1):419. doi: 10.1007/s10143-026-04341-7 (PMC13198487; doi:10.1007/s10143-026-04341-7)
Supplement: Supplementary file 1 — Supplementary Material 1 [file 10143_2026_4341_MOESM1_ESM.docx]

Supplementary Tables

| SDH Evacuation Procedure Codes |
| --- |
| 00C10ZZ, 00C13ZZ, 00C30ZZ, 00C33ZZ, 00C40ZZ, 00C43ZZ, 00C00ZZ, 00C03ZZ, 00C60ZZ, 00C70ZZ, 00CC0ZZ, 00CC3ZZ, 00B00ZZ, 00C20ZZ, 00C40ZZ, 00C43ZZ, 00N00ZZ, 00N10ZZ, 00N20ZZ, 00N60ZZ, 00N70ZZ, 00N80ZZ, 00N90ZZ, 00NA0ZZ, 00NB0ZZ, 00NC0ZZ, 00ND0ZZ, 00U207Z, 00U20JZ, 00U20KZ, 00U237Z, 00U23JZ, 00U23KZ, 00C50ZZ, 00C80ZZ, 00C90ZZ, 00CA0ZZ, 00CB0ZZ, 00CC0ZZ, 00CD0ZZ, 00Q00ZZ, 00Q10ZZ, 00Q20ZZ, 00Q60ZZ, 00Q70ZZ, 00Q80ZZ, 00Q90ZZ, 00QA0ZZ, 00QB0ZZ, 00QC0ZZ, 00QD0ZZ, 009130Z, 00913ZX, 00913ZZ, 009140Z, 00914ZX, 00914ZZ, 009300Z, 009330Z, 00933ZZ, 009340Z, 00934ZZ, 009430Z, 00943ZZ |

Supplementary Table 1: ICD-10 codes for SDH evacuation.

| **Outcome** | | **All** | | **Non-ICP** | | **ICP** | | ***P* value** |
| --- | --- | --- | --- | --- | --- | --- | --- | --- |
| ICU length of stay, days, median (IQR) |  | 10 | (5, 18) | 8 | (4, 15) | 15 | (7, 23) | <0.001 |
| Length of stay, days, median (IQR) |  | 15 | (6, 28) | 12 | (5, 24) | 20 | (9, 36) | <0.001 |
| ICP hours (median,IQR) | |  |  |  |  | 3.52 | (2.08, 8.08) |  |
| Emergency department disposition | Operating room | 2306 | 58.6% | 1562 | 63.7% | 744 | 50.2% | <0.001 |
|  | ICU | 1522 | 38.7% | 809 | 33.0% | 713 | 48.1% | <0.001 |
| Hospital disposition | Home/routine | 279 | 7.1% | 192 | 7.8% | 87 | 5.9% | 0.024 |
|  | Inpatient rehab | 863 | 21.9% | 522 | 21.3% | 341 | 23.0% | 0.22 |
|  | Care facility or other hospitals | 1091 | 27.7% | 653 | 26.6% | 438 | 29.6% | 0.051 |
| Died in hospital | | 1641 | 41.7% | 1045 | 42.6% | 596 | 40.2% | 0.15 |

Supplementary Table 2: Patient outcomes before propensity score matching

| **Demographic** | | **All** |  | **nonICP** |  | **ICP** |  | ***P* value** |
| --- | --- | --- | --- | --- | --- | --- | --- | --- |
|  |  | **n = 2306** | | **n=1562** | | **n = 744** | |  |
| Age, median(IQR) | | 45 | (30, 63) | 50 | (33, 67) | 37 | (27, 54) | <0.001 |
| Age group | 18-35 | 599 | 26.0% | 334 | 21.4% | 265 | 35.6% |  |
|  | 36-50 | 384 | 16.7% | 240 | 15.4% | 144 | 19.4% |  |
|  | 51-65 | 336 | 14.6% | 213 | 13.6% | 123 | 16.5% |  |
|  | >65 | 987 | 42.8% | 775 | 49.6% | 212 | 28.5% |  |
| BMI, median(IQR) | | 26.2 | (23.0, 30.2) | 26.2 | (23.0, 30.3) | 26.1 | (23.1, 30.0) | 0.49 |
| ISS, median(IQR) | | 29 | (26, 36) | 27 | (26, 35) | 30 | (26, 38) | <0.001 |
| ISS severity | <=25 (modirate to severe) | 225 | 9.8% | 163 | 10.4% | 62 | 8.3% |  |
|  | 26-30 (very severe) | 1405 | 60.9% | 1003 | 64.2% | 402 | 54.0% |  |
|  | >30 Critical) | 675 | 29.3% | 395 | 25.3% | 280 | 37.6% |  |
| GCS, median(IQR) | | 3 | (3, 5) | 3 | (3, 6) | 3 | (3, 5) | 0.44 |
| GCS level | 3 | 1527 | 66.2% | 1026 | 65.7% | 501 | 67.3% |  |
|  | 4-6 | 464 | 20.1% | 322 | 20.6% | 142 | 19.1% |  |
|  | 7-8 | 315 | 13.7% | 214 | 13.7% | 101 | 13.6% |  |
| Gender | Male | 1725 | 74.8% | 1138 | 72.9% | 587 | 78.9% | 0.002 |
|  | Felame | 575 | 24.9% | 419 | 26.8% | 156 | 21.0% |  |
| Race | White | 1534 | 66.5% | 1061 | 67.9% | 473 | 63.6% | 0.038 |
|  | non-White | 772 | 33.5% | 501 | 32.1% | 271 | 36.4% |  |
| Ethnicity | Hispanic or Latino | 381 | 16.5% | 234 | 15.0% | 147 | 19.8% | 0.004 |
| Transfusion | | 967 | 41.9% | 606 | 38.8% | 361 | 48.5% | <0.001 |
| Pre-Hospital Cardiac Arrest | | 83 | 3.6% | 51 | 3.3% | 32 | 4.3% | 0.21 |
| Trauma center level | Level 1 | 1352 | 58.6% | 878 | 56.2% | 474 | 63.7% | <0.001 |
|  | other | 954 | 41.4% | 684 | 43.8% | 270 | 36.3% |  |
| Mechanism of Injury | Fall | 1028 | 44.6% | 808 | 51.7% | 220 | 29.6% | <0.001 |
|  | MVT/Transport accidents | 1128 | 48.9% | 658 | 42.1% | 470 | 63.2% |  |
|  | Struck by and other | 150 | 6.5% | 96 | 6.1% | 54 | 7.3% |  |
| Bedsize | Small/Medium (<=600) | 1405 | 60.9% | 925 | 59.2% | 480 | 64.5% | 0.015 |
|  | Large Hospital(> 600) | 901 | 39.1% | 637 | 40.8% | 264 | 35.5% |  |
| Hospital Teaching Status | Academic/university | 1227 | 53.2% | 786 | 50.3% | 441 | 59.3% | <0.001 |
|  | Community/Nonteaching | 1073 | 46.5% | 772 | 49.4% | 301 | 40.5% |  |
| Hospital Type | For Profit | 260 | 11.3% | 194 | 12.4% | 66 | 8.9% | 0.012 |
|  | Non-profit/government | 2046 | 88.7% | 1368 | 87.6% | 678 | 91.1% |  |
| Payment | Private/Commercial Insurance | 848 | 36.8% | 526 | 33.7% | 322 | 43.3% | <0.001 |
|  | Medicare | 513 | 22.2% | 437 | 28.0% | 76 | 10.2% |  |
|  | Medicaid | 507 | 22.0% | 308 | 19.7% | 199 | 26.7% |  |
|  | Self pay and Other | 438 | 19.0% | 291 | 18.6% | 147 | 19.8% |  |
| ICU length of stay, days, median (IQR) |  | 9 | (4, 16) | 7 | (3, 14) | 12 | (6, 21) | <0.001 |
| Length of stay, days, median (IQR) |  | 12 | (5, 25) | 11 | (4, 23) | 16 | (7, 32) | <0.001 |
| ICP hours (median,IQR) | |  |  |  |  | 2.72 | (1.75, 6.22) |  |
| Hospital disposition | Home/routine | 162 | 7.0% | 121 | 7.7% | 41 | 5.5% | 0.049 |
|  | Inpatient rehab | 467 | 20.3% | 312 | 20.0% | 155 | 20.8% | 0.63 |
|  | Care facility or other hospitals | 597 | 25.9% | 402 | 25.7% | 195 | 26.2% | 0.81 |
| Died in hospital | | 1052 | 45.6% | 705 | 45.1% | 347 | 46.6% | 0.50 |

Supplementary Table 3: Pre-matched subgroup analysis patient characteristics and outcomes.

| **Demographic** | | **nonICP** |  | **ICP** |  | ***P* value** |
| --- | --- | --- | --- | --- | --- | --- |
|  |  | **n=665** | | **n = 665** | |  |
| Age, median(IQR) | | 39 | (27, 58) | 39 | (27,55) | 0.42 |
| Age group | 18-35 | 227 | 34.1% | 219 | 32.9% |  |
|  | 36-50 | 130 | 19.5% | 128 | 19.2% |  |
|  | 51-65 | 97 | 14.6% | 119 | 17.9% |  |
|  | >65 | 211 | 31.7% | 199 | 29.9% |  |
| BMI, median(IQR) | | 25.9 | (22.9, 30.1) | 26.1 | (22.9, 30.0) | 0.99 |
| ISS, median(IQR) | | 30 | (26, 38) | 30 | (26, 38) | 0.53 |
| ISS severity | <=25 (moderate to severe) | 50 | 7.5% | 61 | 9.2% | 0.45 |
|  | 26-30 (very severe) | 353 | 53.1% | 368 | 55.3% |  |
|  | >30 Critical) | 262 | 39.4% | 236 | 35.5% |  |
| GCS, median(IQR) | | 3 | (3, 5) | 3 | (3, 5) | 0.73 |
| GCS level | 3 | 450 | 67.7% | 446 | 67.1% |  |
|  | 4-5 | 134 | 20.2% | 130 | 19.5% |  |
|  | 6-8 | 81 | 12.2% | 89 | 13.4% |  |
| Gender | Male | 526 | 79.1% | 520 | 78.2% | 0.69 |
|  | Female | 139 | 20.9% | 145 | 21.8% |  |
| Race | White | 459 | 69.0% | 437 | 65.7% | 0.20 |
|  | non-White | 206 | 31.0% | 228 | 34.3% |  |
| Ethnicity | Hispanic or Latino | 109 | 16.4% | 125 | 18.8% | 0.25 |
| Transfusion | | 319 | 48.0% | 314 | 47.2% | 0.78 |
| Pre-Hospital Cardiac Arrest | | 42 | 6.3% | 41 | 6.2% | 0.91 |
| Trauma center level | Level 1 | 418 | 62.9% | 420 | 63.2% | 0.91 |
|  | other | 247 | 37.1% | 245 | 36.8% |  |
| Mechanism of Injury | Fall | 212 | 31.9% | 212 | 31.9% | 1.00 |
|  | MVT/Transport accidents | 420 | 63.2% | 420 | 63.2% |  |
|  | Struck by and other | 33 | 5.0% | 33 | 5.0% |  |
| Bedsize | Small/Medium (<=600) | 406 | 61.1% | 421 | 63.3% | 0.40 |
|  | Large Hospital(> 600) | 259 | 38.9% | 244 | 36.7% |  |
| Hospital Teaching Status | Academic/university | 392 | 58.9% | 392 | 58.9% | 1.00 |
|  | Community/Nonteaching | 273 | 41.1% | 273 | 41.1% |  |
| Hospital Type | For Profit | 76 | 11.4% | 58 | 8.7% | 0.10 |
|  | Non-profit/government | 589 | 88.6% | 607 | 91.3% |  |
| Payment | Private/Commercial Insurance | 240 | 36.1% | 289 | 43.5% | <0.001 |
|  | Medicare | 123 | 18.5% | 72 | 10.8% |  |
|  | Medicaid | 169 | 25.4% | 173 | 26.0% |  |
|  | Other | 133 | 20.0% | 131 | 19.7% |  |

Supplementary Table 4: Subgroup analysis patient characteristics.
